# Response of Leaf Traits of Eastern Qinghai-Tibetan Broad-Leaved Woody Plants to Climatic Factors

**DOI:** 10.3389/fpls.2021.679726

**Published:** 2021-07-30

**Authors:** Xiaomei Kang, Yanan Li, Jieyang Zhou, Shiting Zhang, Chenxi Li, Juhong Wang, Wei Liu, Wei Qi

**Affiliations:** ^1^State Key Laboratory of Grassland Agro-ecosystems, School of Life Sciences, Lanzhou University, Lanzhou, China; ^2^College of Life Science and Food Technology, Hanshan Normal University, Chaozhou, China

**Keywords:** climate, functional tradeoff, leaf morphology, leaf stoichiometry pattern, Qinghai-Tibet Plateau, specific leaf area, stomatal density, stomatal pore area index

## Abstract

Plant ecologists have long been interested in quantifying how leaf traits vary with climate factors, but there is a paucity of knowledge on these relationships given a large number of the relevant leaf traits and climate factors to be considered. We examined the responses of 11 leaf traits (including leaf morphology, stomatal structure and chemical properties) to eight common climate factors for 340 eastern Qinghai-Tibetan woody species. We showed temperature as the strongest predictor of leaf size and shape, stomatal size and form, and leaf nitrogen and phosphorus concentrations, implying the important role of local heat quantity in determining the variation in the cell- or organ-level leaf morphology and leaf biochemical properties. The effects of moisture-related climate factors (including precipitation and humidity) on leaf growth were mainly through variability in leaf traits (e.g., specific leaf area and stomatal density) related to plant water-use physiological processes. In contrast, sunshine hours affected mainly cell- and organ-level leaf size and shape, with plants developing small/narrow leaves and stomata to decrease leaf damage and water loss under prolonged solar radiation. Moreover, two sets of significant leaf trait-climate relationships, i.e., the leaf/stomata size traits co-varying with temperature, and the water use-related leaf traits co-varying with precipitation, were obtained when analyzing multi-trait relationships, suggesting these traits as good indicators of climate gradients. Our findings contributed evidence to enhance understanding of the regional patterns in leaf trait variation and its environmental determinants.

## Introduction

The relationship between plants and their living environment is one of the core issues in plant ecology (Hedin, 2004; Wright et al., 2017). Leaf is the main plant organ exposed directly to atmospheric environment, and thus, expected and observed to vary with climatic gradients (Reich and Oleksyn, 2004; Wright et al., 2005; Maire et al., 2015; Gong et al., 2020). Leaf traits can be manifested in many types (e.g., morphology, stomatal structure, stoichiometry, and physiology) or levels (e.g., cell, tissue, and organ), and generally show different responses to climate (McDonald et al., 2003; Ordoñez et al., 2009; Meng et al., 2015; Yang et al., 2019). Consequently, quantifying and comparing the responses of different types’ or levels’ leaf traits to climate factors should be important for a thorough understanding of how plants adapt to climate change and drive community assembly (Ackerly et al., 2002; Wright et al., 2005; Maire et al., 2015; Gong et al., 2018; Yang et al., 2019).

Leaf morphology, including the length, size, shape, and thickness of leaf, affects directly plants’ ability for light interception and carbon acquisition (Milla and Reich, 2007; Peppe et al., 2011; Wright et al., 2017). The mechanism of leaf morphological formation in relation to adaptive value is generally believed to be related to a change in the balance of energy or water of the leaf (Niinemets et al., 2006; Peppe et al., 2011; Maire et al., 2015; Meng et al., 2015). Typically, large and (or) broad leaves predominate in cool, humid, or shady environments because their thicker leaf margins induce greater resistance to transport of heat and substances (Ackerly et al., 2002; McDonald et al., 2003; Niinemets et al., 2006; Leigh et al., 2017), but small and (or) narrow leaves are considered advantageous in hot, dry, and high light habitats due to their ability of increasing leaf heat exchange, avoiding leaf damage, and maintaining leaf water content (Ackerly et al., 2002; Bragg and Westoby, 2002; Peppe et al., 2011). However, leaf size has also been found to decrease with temperature or irradiance gradient because low light and short growing season make against leaf carbon acquisition (McDonald et al., 2003; Wright et al., 2005, 2017; Meng et al., 2015). Specific leaf area (SLA, ratio of leaf area to leaf dry mass) is a commonly used index highly negatively related to leaf thickness. SLA is tightly associated with leaf physiological processes related to water use, e.g., photosynthesis and transpiration, and is sensitive to moisture conditions (e.g., precipitation, humidity, and soil water; Gouveia and Freitas, 2009; Dwyer et al., 2014; Wilcox et al., 2021).

Stoma, the main channels for exchanging water and CO_2_, controls directly the photosynthesis, transpiration, and respiration (Franks and Beerling, 2009; Yan et al., 2017; Harrison et al., 2020). Of stomatal traits, stomatal size, shape, and density are the most common measurements to assess plant’s response to environmental gradients (Liu et al., 2018; Bertolino et al., 2019; Harrison et al., 2020). Stomatal size and shape are highly associated with maximum stomatal conductance, determining leaf maximum photosynthetic assimilation and gas exchange capacity, whereas stomatal density is inversely proportional to the distance that gas molecules have to diffuse through the stomatal pore, determining the speed, and flux of stomatal gas exchange (Liu et al., 2018; Bertolino et al., 2019). In high-temperature and especially high-moisture habitats, large or broad-round stomata and high stomatal density are expected in response to the increase of leaf transpiration and respiration (Yan et al., 2017; Liu et al., 2018; Bertolino et al., 2019). However, to maintain an appropriate proportion between stomatal guard cells and other epidermal cells, the total stomatal area is limited. Thus, developing fewer, larger stomata versus more, smaller stomata often follows a fundamental tradeoff (Franks and Beerling, 2009; Bucher et al., 2016; Liu et al., 2018). A few studies have reported that the tradeoff is susceptible to environments and plant species, which led to the often conflicting findings about the stomatal trait-climate relationship (Franks and Beerling, 2009; Liu et al., 2018).

Nitrogen (N) and phosphorus (P) are generally considered the two most limiting elements affecting leaf physiological processes and biochemical activities (e.g., enzyme activity and protein synthesis; Reich and Oleksyn, 2004; Niinemets et al., 2006; Ordoñez et al., 2009). Climatic factors can affect these processes/activities, and thus leaf N and P concentration (Wright et al., 2005; Wang et al., 2015; Yan et al., 2017). For two decades now, many large-scale studies have demonstrated that, to keep stable ability of photosynthetic carbon gain, plants increase their leaf N and P concentration with decreasing temperature and (or) available water to compensate for the decreasing in metabolic rate and the activity of N-rich enzymes and P-rich RNA (Niinemets, 2001; Han et al., 2005; Ordoñez et al., 2009; Wang et al., 2015; Gong et al., 2018). However, other studies that focused on regional or local trait variation often found opposite results (He et al., 2008; Zhao et al., 2014), suggesting that some critical questions of the leaf stoichiometry patterns and their determinants have not been fully revealed.

To sum up, there is still no consistent pattern how leaf traits vary with climate. This may be caused by two reasons. Firstly, leaf traits generally do not vary independently, but show significant patterns of covariation due to functional tradeoffs or genetic controls (Niinemets, 2001; Yang et al., 2019). They often form a “syndrome,” as exemplified by the leaf economics spectrum (LES; Niinemets, 2001; Wright et al., 2005; Yang et al., 2019), to adapt to environments coordinatedlly (Wright et al., 2005). Thus, the leaf trait–climate relationship may be obscured by the covariation among traits. Then, the pattern of leaf trait variation is the result of the combined effect of multiple climatic factors, but the geographic variation in climatic factors differs among regions. In this case, the response of single leaf trait to a certain factor may be different among study regions. Thus, researches quantifying the leaf trait–climate relationship in a multi-trait and multi-factor space are crucial to examine plants’ local adaptation and regional distribution, but have received little attention (Wright et al., 2005; Maire et al., 2015; Meng et al., 2015). In the study, we assembled a database of eleven leaf traits of 340 angiosperm species (641 populations) from 46 sites of an eastern part of the Qinghai-Tibet Plateau (QTP). These sites are overall cool or cold, and span a wide temperature range (mean annual temperature, 0 – 12°C). In contrast, all sites are located in the semi humid area (mean annual precipitation, 520–690 mm), suggesting small among-site difference in precipitation and possibly low water limitation on species distribution and plant organ growth. We aimed to examine the relative contributions of eight common climate factors in leaf trait variation at the regional scale. These leaf traits include leaf morphology, stomatal structure and chemical composition, and thus, can together capture many functions of plants. Specifically, we addressed the following two questions:

(1) How did the leaf traits vary with climate, and what were the chief climatic factors affecting leaf morphology, stomatal traits and chemical composition, respectively?
(2) How many leaf trait combinations, i.e., the sets of significant covarying leaf traits, could be extracted to clearly describe leaf functional responses to climate?

To answer these questions, both univariate and multivariate linear models and redundancy analysis (RDA) were used. Based on these analyses, we expected (1) that temperature should be the strongest predictor of the variation of most leaf traits in the cool QTP zone where local heat quantity is general the limiting factor of plant individual and organ growth, (2) that, due to possibly low water limitation on plant organ growth, moisture-related climate factors, including precipitation and humidity, should exert less impact than temperature on the variation of most leaf traits except for the traits related to water-use physiological processes (e.g., SLA and some of stomatal traits), and (3) that, because the covariation among leaf traits is universal, plants should form several sets of leaf trait combinations to adapt to different climate factors. Besides, we could not expect the pattern of leaf trait variation with sunshine hour due to the lack of relevant multi-species research. We believe that the study can provide a clear understanding of regional pattern of leaf trait variation and its climate determinants.

## Materials and Methods

### Study Sites and Field Sampling

The study area is located on the eastern edge of Tibetan Plateau (101°05′–104°20′ E, 33°25′–35°30′ N, about 40,000 km^2^), and belong to a transitional region of semi-humid and semi-arid areas. In this region, altitude is the strongest determinant of bioclimatic gradients (Table 1), where with increasing altitude, climatic vary from north-subtropics, warm-temperate, cool-temperate, subalpine to alpine, and vegetations from broad-leaved forest, coniferous and broad-leaved mixed forest to shrub.

**TABLE 1:** A comparison of climate and vegetation characteristics of each 300-m altitudinal belt.

Altitudinal belt (m)	Climatic zone	Zonal woody vegetation type	MAT (°C)	FFP (days)	LGS (days)
1,600–1,900	North-subtropical—warm temperate	Broadleaf forest	11–13	200–240	230–270
1,900–2,200	Warm temperate	Broadleaf forest	8–11	160–210	210–250
2,200–2,500	Warm temperate	Broadleaf forest	6–9	120–170	190–230
2,500–2,800	Cool temperate	Mixed forest	4–7	80–130	170–210
2,800–3,100	Cool temperate—subalpine	Mixed forest-Shrub	3–5	40–90	150–190
3,100–3,400	Subalpine	Mixed forest-Shrub	1–4	0–50	140–170
3,400–3,700	Subalpine—alpine	Shrub	0–3	0–20	120–160
3,700–4,000	Alpine	Shrub	−2 to 1	0	100–140

*MAT, mean annual temperature; FFP, (absolute) frost-free period; LGS, length of the growing season (based on the average phenological performance of the local dominant woody species); Mixed forest, mixed conifer-deciduous broadleaf forest.*

*The climatic zone or vegetation type of each altitudinal belt is a rough classification frame because altitudinal variation in climatic and vegetation is transitional.*

**FIGURE 1:**
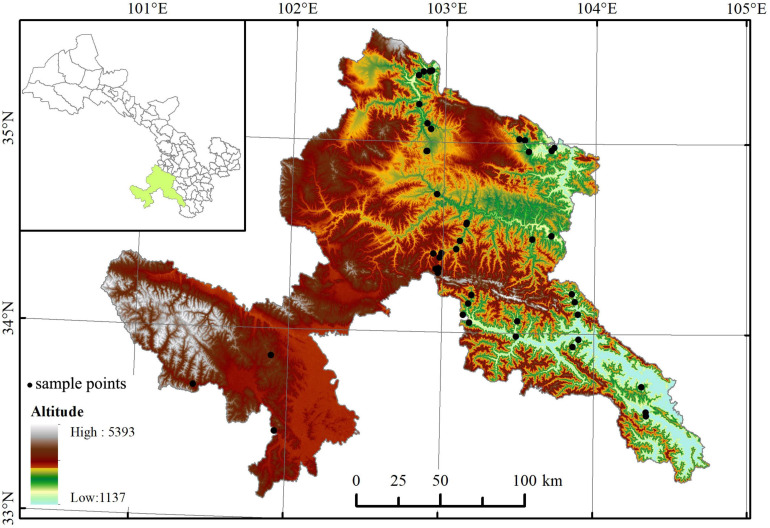
Distribution of the 46 study sites across the eastern Qinghai-Tibetan vegetation zone. The map was edited and generated with ArcGIS 10.2 software, http://www.esri.com/.

Field sampling was carried out from June to September of 2018 and 2019. In each 300 m altitudinal belt (Table 1), four to nine 300 m × 300 m sites (altogether 46 sites, with elevation ranging from 1,758 m to 3,924 m a.s.l., seen in Supplementary Material) were set up (less sites in alpine belts for less woody species number, smaller woody vegetation area, and lower among-site difference in species composition). We recorded the altitude, latitude, and longitude of each site (Figure 1). A total of 641 populations of 340 species, belonging to 98 genera of 46 families (according to the Angiosperm Phylogeny Group IV classification system, undated in 2016), were collected. For each population, 3–5 individuals that grew well (i.e., mature and living in the habitat with sufficient nutrition and low disturbance) were selected. Then, for each individual, 2–3 branches with 5–20 mature, healthy, finished expansion, and unbroken leaves on each branch were chosen at random at the outer canopy for the purpose of avoiding obvious difference in light condition (Gagliardi et al., 2015). The mixed leaves from different individuals were brought indoors with a portable refrigerator.

### Leaf Traits

For each population, we measured eleven leaf traits, including four leaf morphological traits, i.e., leaf length (LL), leaf area (LA), specific leaf area (SLA,1) and leaf length-width ratio (LL/LW), four stomatal traits, i.e., stomatal density (SD, 2), stomatal length-width ratio (SL/SW), stomatal area (SA, 3) and stomatal pore area index (SPI, 4; Bucher et al., 2016), and three leaf chemical traits, i.e., leaf nitrogen per mass (N_mass_), leaf phosphorus per mass (P_mass_), and leaf nitrogen-phosphorus ratio (N/P).

(1) $$S\,p\,e\,c\,i\,f\,i\,c\,l\,e\,a\,f\,a\,r\,e\,a=\frac{l\,e\,a\,f\,a\,r\,e\,a}{l\,e\,a\,f\,d\,r\,y\,m\,a\,s\,s}$$

(2) $$S\,t\,o\,m\,a\,t\,a\,l\,d\,e\,n\,s\,i\,t\,y=\frac{s\,t\,o\,m\,a\,t\,a\,l\,n\,u\,m\,b\,e\,r\,i\,n\,v\,i\,e\,w\,a\,r\,e\,a}{v\,i\,e\,w\,a\,r\,e\,a}$$

(3) $$s\,t\,o\,m\,a\,t\,a\,l\,a\,r\,e\,a=\frac{\pi }{4}\times s\,t\,o\,m\,a\,t\,a\,l\,l\,e\,n\,g\,t\,h\times s\,t\,o\,m\,a\,t\,a\,l\,w\,i\,d\,t\,h$$

(4) S⁢t⁢o⁢m⁢a⁢t⁢a⁢l⁢p⁢o⁢r⁢e⁢a⁢r⁢e⁢a⁢i⁢n⁢d⁢e⁢x=s⁢t⁢o⁢m⁢a⁢t⁢a⁢l⁢d⁢e⁢n⁢s⁢i⁢t⁢y×s⁢t⁢o⁢m⁢a⁢t⁢a⁢l⁢a⁢r⁢e⁢a

For measuring leaf morphological traits, 5–20 leaves (or 2–4 large leaves) from different individuals of a population were bulked together representing one replicate. LA of each population was determined by scanning the leaves with a flatbed scanner (HP Laser Jet 1320) with 3–4 replicates and analyzing the pictures with image analysis software (Image J)^1^. In each replicate, all selected leaves were placed on scanner to avoid overlap and fully expand bent or contracted leaves. For each replicate, all imaged leaves were then dried at 65°C to a constant mass and weighed to the nearest 0.0001 g. Leaf length and width for each population were also determined by analyzing scanned pictures (8–15 leaves were randomly selected), in which LL was measured from lamina tip to the intersection of the lamina and petiole along the lamina midrib, and leaf width (LW) was measured from tip to tip between the widest lamina lobes. For stomatal traits, three or five fully expanded leaves per population were randomly selected for stomatal observation based on the abaxial (lower) surface by the nail polish impression method (Franks and Beerling, 2009; Wang et al., 2019). The stomatal traits were measured using a Leica DFC 450 camera (Nussloch, Germany) mounted on a Leica DM 2500 microscopeat at 400× magnification. Stomatal length (SL, μm) and stomatal width (SW, μm) were measured as the guard cell length and guard cell pair width based on 45 stomata per population (15 stomata × 3 leaves or 9 stomata × 5 leaves). Stomatal density (number × mm^–2^) was calculated as the number of stomata per unit of epidermal surface based on 30 fields of view per population (10 fields × 3 leaves or 6 fields × 5 leaves). Leaf N and P were determined by drying leaves at 70°C, 0.100 g of leaf dry mass after grounding them into a fine powder, and their concentration were then measured by using an elemental analyzer after digesting with H_2_SO_4_.

### Climate Data

We selected eight frequently used climatic factors, including MAT, mean temperature of the warmest quarter (MTWQ, °C) and the coldest quarter (MTCQ, °C), mean annual sunshine hours (MASH), MAP, precipitation of the wettest quarter (PWQ, mm) and the driest quarter (PDQ, mm) and mean relative humidity (MRH, %). For each sampling site, data for each climatic variable were obtained from the National Meteorological Bureau of China database of monthly records from 22 climate stations across study area^2^ and represented by Co Kriging interpolation using ArcGIS software version 10.6 (ESRI, Redlands, CA, United States).

Due to the possibly high correlation among climate factors, we conducted principal component analyses (PCA) on all climate variables to address multicollinearity and obtain the main climate predictors for leaf trait variation in multivariable linear analysis. Results showed that three PC axes explained >90% of the total climate variance. The variables (MTWQ, PDQ, and MRH) with the largest load on each of the three axes were initially selected as the key climate predictors (Supplementary Appendix 1). We then examined the correlation matrix of variables, and kept the variable (MASH) with weak correlation (r < 0.7) to the key predictors to avoid missing significant predictors (Dormann et al., 2013). The four selected main climate predictors, i.e., MTWQ, PDQ, MRH, and MASH, represent separately site’s temperature, precipitation, humidity, and sunshine time.

### Data Analysis

Data on LL, LL/LW, LA, SLA, SD, and SA were log-transformed before analyses in order to fit a normal distribution. We firstly used simple linear regression to examine the effect of each eight climatic factors on each of eleven leaf traits. We then fitted multiple linear regressions (MLRs) to investigate the multivariable effect of four main climate predictors (MTWQ, PDQ, MRH, and MASH) on each leaf trait (response variable). After initial model fit, a stepwise (bidirectional elimination) model selection routine was used to choose the model with the minimum Akaike information criterion (AIC) value for each trait. Finally, a redundancy analysis (RDA) was conducted with eleven leaf traits as the response variables, and eight climate variables as explanatory variables to assess the multivariate relationships between leaf traits and climatic factors. The RDA analysis was performed using “vegan” package in R 3.0.2.

## Results

Comparing among three-type leaf traits, stronger climatic effect was found on leaf chemical traits than on other leaf traits, in which the effect of temperature variables (MAT, MTWQ, MTCQ) on P_mass_ and N_mass_ was significantly negative, but on N/P was significantly positive. The effect of other climate variables, however, was generally positive on P_mass_ and N_mass_, but negative on N/P (Table 2). Moreover, temperature variables and sunshine hour (MASH) were the main factors affecting leaf stomatal and morphological traits except for SLA and SD, in which the effect of temperature variables on LL, LA, SA, and SPI was significantly positive, but on LL/LW and SL/SW was significantly negative. By contrast, the effect of MASH on LL/LW and SL/SW was positive, but on LL, LA, and SA was negative. SLA was affected mainly by moisture-related climatic factors including precipitation variables (MAP, PWQ, and PDQ) and humidity (MRH). The response of SD, however, was generally nonsignificant to climatic factors except for PWQ and PDQ (marginally significant positive response; Table 2).

**TABLE 2:** Results of the linear relationship between leaf traits and climate factors.

	MAT	MTWQ	MTCQ	MAP	PWQ	PDQ	MASH	MRH
LL	0.458***	0.450***	0.428***	–0.025	−0.126**	0.040	−0.240***	−0.097**
LL/LW	−0.084*	−0.073*	−0.090*	–0.041	0.004	–0.042	0.109**	0.030
LA	0.474***	0.464***	0.446***	–0.013	−0.128**	0.052	−0.273***	−0.103**
SLA	–0.009	0.020	–0.054	−0.084*	−0.185***	−0.184***	0.053	0.122**
SD	–0.032	–0.048	–0.005	0.068	0.105*	0.093*	–0.038	–0.005
SL/SW	−0.126**	−0.112**	−0.134***	–0.051	–0.020	−0.079*	0.120**	0.025
SA	0.141***	0.134***	0.139***	0.008	–0.011	0.062	−0.118**	−0.072*
SPI	0.116***	0.124***	0.133***	0.055	0.063	0.098**	−0.143***	−0.069*
P_mass_	−0.439***	−0.465***	−0.375***	0.208***	0.262***	0.139**	0.111**	0.185***
N_mass_	−0.347***	−0.354***	−0.311***	0.095**	0.127**	–0.025	0.127**	0.114**
N/P	0.293***	0.315***	0.242***	−0.168***	−0.235***	−0.158***	−0.096*	−0.163***

*For each relationship, standard regression coefficient and its significance are shown.*

LL, leaf length; LL/LW, leaf length-width ratio; LA, leaf area; SLA, specific leaf area; SD, stomatal density; SL/SW, stomatal length-width ratio; SA, stomatal area; SPI, stomatal pore area index; N_*mass*_ and P_*mass*_, leaf nitrogen and phosphorus per mass; N/P, leaf nitrogen-phosphorus ratio; MAT, MTWQ and MTCQ, mean temperature of annual, the warmest and coldest quarter; MAP, PWQ and PDQ, precipitation of annual, the wettest and driest quarter; MASH, mean annual sunshine hours; MRH, mean relative humidity.

*P < 0.05.

**P < 0.01.

***P < 0.001.

Result of MLRs showed that four selected main climate variables explained a large proportion of variation in leaf size and chemical traits, but a small proportion of variation in stomatal and other traits (Table 3). Comparing among climatic variables, temperature (MTWQ) had the greatest influence on the variation of leaf traits, which was the strongest predictor of eight leaf traits, including leaf size and shape (LL, LA, and SL/SW), stomatal area and shape (SA and SPI) and leaf chemical traits (N_mass_, P_mass_, and N/P). MASH was the strongest predictor of LL/LW and the significant predictor of other seven leaf traits (LL, LA, SLA, SL/SW, SA, P_mass_, and N/P). PDQ was the strongest predictor of SD and SLA and the significant predictor of other three leaf traits (SPI, P_mass_, and N/P), while MRH was only the significant predictor of three leaf morphological traits (LL, LA, and SLA).

**TABLE 3:** Results of multiple linear regressions (MLRs, stepwise model selection routine) for the effects of four main climate predictors on each of eleven leaf traits.

	AIC	R^2^	*P*
LL = −0.770 + 0.053**MTWQ** − 0.0004MASH + 0.025MRH	–1764.44	0.222	<0.001
LL/LW = −0.409 + 0.0003**MASH**	–1899.05	0.010	0.007
LA = −1.752 + 0.108**MTWQ** − 0.001MASH + 0.055MRH	–894.01	0.242	<0.001
SLA = 2.415−0.029**PDQ** − 0.0004MASH + 0.017MRH	–2238.05	0.063	<0.001
SD = 2.233 + 0.011**PDQ**	–1778.69	0.007	0.027
SL/SW = 0.840 − 0.010**MTWQ** + 0.0004MASH	–1369.42	0.017	0.003
SA = 2.972 + 0.012**MTWQ** − 0.0003MASH	–1525.42	0.020	<0.001
SPI = 4.454 + 0.011**MTWQ** − 0.020PDQ	–1495.59	0.022	<0.001
P_mass_ = 0.174 − 0.023**MTWQ** + 0.014PDQ + 0.0002MASH	–2692.53	0.233	<0.001
N_mass_ = 8.095 − 0.189**MTWQ**	238.20	0.124	<0.001
N/P = 38.743 + 0.340**MTWQ** − 0.577PDQ − 0.010MASH	0.48	0.136	<0.001

*For each regression equation, significant climatic predictors are arranged in order of their importance (explanatory power for leaf trait variation; R^2^), with the strongest predictor (marked in bold) on the leftmost of the equation. Abbreviations of leaf traits and climatic factors are as specified in Table 2.*

**FIGURE 2:**
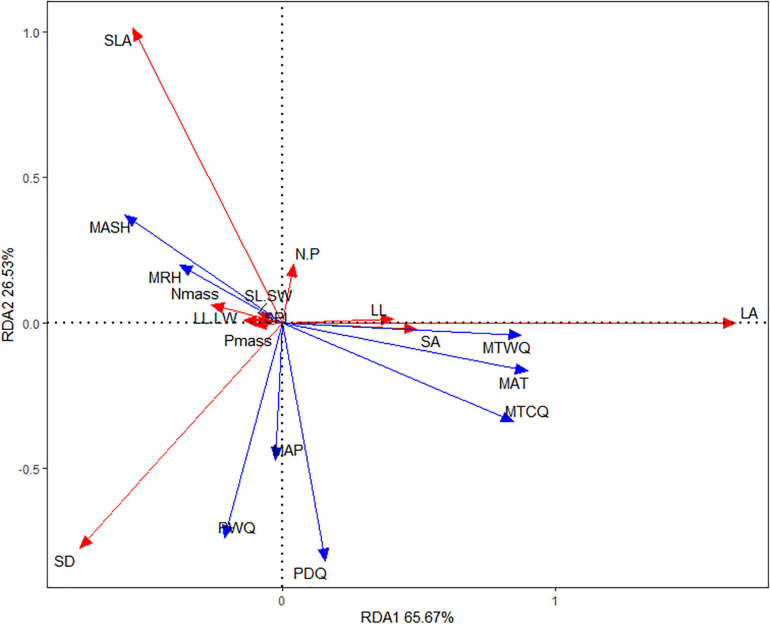
Climate-related leaf trait dimensions from redundancy analysis (RDA). Red and blue arrows represent leaf traits and climatic factors, respectively. LL.LW, leaf length-width ratio; SL.SW, stomatal length-width ratio; Nmass, leaf nitrogen per mass; Pmass, leaf phosphorus per mass; N.P, leaf nitrogen-phosphorus ratio. Abbreviations of climate factors and part of leaf traits are as specified in Table 2.

Redundancy analysis revealed the effects of eight climatic factors on leaf traits, in which RDA1 and RDA2 accounted for 65.67 and 26.53%, respectively, of the changes of leaf traits (Figure 2). RAD1, explaining mainly the variation in LA, LL, and SA (positive relationship) and N_mass_ (negative relationship), was strongly positively correlated with temperature variables (MAT, MTCQ, and MTWQ), but weakly negatively correlated with MASH and MRH. RAD2, explaining mainly the variation in SLA and N/P (positive relationship) and SD (negative relationship), was strongly negatively correlated with precipitation variables (PWQ, PDQ, and MAP). Moreover, in the bidimensional ordering chart of the RDA, temperature variables predicted best the variation of LA, LL, and SA, but precipitation variables predicted best the variation of SLA and SD. Besides, MASH and MRH predicted partly N_mass_ variation, and precipitation predicted partly leaf N/P variation.

## Discussion

We show a significant effect of climate factors on leaf trait variations. The effect, however, is overall modest, which is consistent with most previous regional or larger scale multi-species studies (Wright et al., 2005, 2017; He et al., 2008; Meng et al., 2015; Yang et al., 2019), suggesting that other factors such as soil properties (Ordoñez et al., 2009; Zhao et al., 2014; Maire et al., 2015; Gong et al., 2020) may also contribute to leaf trait variation. Meanwhile, we have not found similar pattern about the responses of leaf morphological, stomatal, and chemical traits to different climatic factors, implying that a series of opposite mechanisms or processes may operate simultaneously in determining local adaptation and regional distribution of leaf traits.

As our first expectation, temperature is the strongest factor affecting positively leaf size traits (LL and LA). The result is opposite to the common findings that leaf size decreases with increasing temperature because the margins of large leaf make against heat transport (Ackerly et al., 2002; McDonald et al., 2003; Niinemets et al., 2006; Leigh et al., 2017). The inconsistence may be that, for the cool QTP zone, the effect of heat stress on leaf development may be nonsignificant for most plants even if they are in warm low altitude. By contrast, plants usually have a long growing season in low altitude, which can afford the growth of large leaves (McDonald et al., 2003; Peppe et al., 2011; Maire et al., 2015; Yang et al., 2019). Meanwhile, large leaves mean a high light competition and interception ability and a high photosynthetic efficiency, which is important for plant survival in shady low altitude where forests are characterized by closed canopy (Ackerly et al., 2002; Bragg and Westoby, 2002; Milla and Reich, 2007; Wright et al., 2017). In extremely cold subalpine/alpine belts, however, small leaves are generally developed to adapt to low heat quantity, and to reduce leaf freezing damage caused by leaf surface transpiration (McDonald et al., 2003; Meng et al., 2015; Wright et al., 2017). Sunshine hour, however, influences significantly negatively leaf size, implying that the QTP woody plants tend to balance between increasing leaf light interception and reducing damage from solar radiation by varying leaf size. Small-leaf plants prefer to live under long solar radiation to maximize their photosynthetic duration time for getting enough nutrition, but large-leaf plants survive chiefly under short sunshine time to minimize leaf damage from leaf light exposure time. Meanwhile, sunshine hour is the only main climate factor affecting (positively) LL/LW (Table 2). This suggests that regional leaf shape varies mainly in response to the amount of solar radiation, rather than it of heat or available water. Besides, the overall weak influence of climatic on LL/LW may imply that other factors, e.g., wind (representing natural drag forces; Vogel, 2009; Louf et al., 2018) or phylogeny (Nicotra et al., 2011), contribute more to the variation of leaf shape. In contrast, the effect of moisture-related climate factors (precipitation and humidity) on leaf morphological traits is generally weak except that they account for a larger proportion of SLA variation than temperature and sunshine hour. The result can be explained by our second expectation that the value of SLA is closely associated with the pattern of water use-related leaf physiology such as photosynthetic efficiency and transpiration rate (Ackerly et al., 2002; Gouveia and Freitas, 2009; Dwyer et al., 2014; Wilcox et al., 2021), and thus, it should be more affected by available water.

We have shown more significant effect of temperature on SA (positive effect) and SL/SW (negative effect), and of precipitation on SD (positive effect). The result may be explained by two reasons. Firstly, in low altitude, for reducing leaf damage from short-term high temperature at noon in summer, plants prefer to develop large and broad-round stomata to maximize instantaneous gas exchange rate because it can ensure a high transient transpiration rate for a fast reduction in leaf surface temperature (Wright et al., 2005; Ordoñez et al., 2009; Liu et al., 2018; Harrison et al., 2020). By comparison, high stomatal density in leaf epidermis, representing short path length for the diffusion of CO_2_ and water vapor and thus high stomatal conductance and transpiration efficiency, is expected common in high-humidity, high-precipitation, or water-rich environments (Franks and Beerling, 2009; Yan et al., 2017; Liu et al., 2018; Bertolino et al., 2019; supporting our second expectation that precipitation affects mainly leaf traits related to plant water-use physiological processes). Surprisingly, the effect of sunshine hour on SA, SL/SW, and SD were significantly negative, significantly positive and nonsignificant, respectively. This indicates that plants generally develop small and narrow stomata but not low stomatal number to reduce leaf transpiration and avoid excessive water loss under long light exposure time. Moreover, SPI shows similar responses as SA but not SD to climatic factors. This reveals that, for the QTP zone, the effect of climate change on leaf gas exchange or metabolism may be mainly regulated by the variation of stomatal size rather than it of stomatal density (Sack et al., 2003; Bucher et al., 2016).

As our expectation, temperature is the strongest factor affecting negatively leaf N or P. This is consistent with most of the findings at global or regional scale, supporting the temperature-plant physiology hypothesis that the increases of N and P concentration in leaves can compensate for the decreases in metabolic rate at low temperature (Niinemets, 2001; Reich and Oleksyn, 2004; Han et al., 2005; Wang et al., 2015; Gong et al., 2018). By comparison, the effect of precipitation or humidity on leaf N or P is weak and generally positive, in which the effect is greater on leaf P than leaf N. Two reasons may contribute to the result. Firstly, all leaf P come from soil, but soil P in natural ecosystem is mainly derived from sedimentary rocks (Ordoñez et al., 2009; Porder and Ramachandran, 2013). Precipitation can accelerate the soil P acquisition from rock parent material (Aerts and Chapin, 2000; Han et al., 2005; Wang et al., 2015) and leaf P uptake from soil (Hedin, 2004; Ordoñez et al., 2009; He et al., 2014), and thus, is expected to increase significantly leaf P concentration. By contrast, due to adequate N supply in atmosphere and multiple mechanisms of N-fixation (Chen et al., 2013), the dependence of leaf N content on precipitation or other environmental factors may be weak. Besides, because N has the property of high diffusivity in soil solution and fast leaching losses from soil, soil N content may increase nonsignificantly with precipitation (Reich and Oleksyn, 2004; He et al., 2008; Chen et al., 2013; Li et al., 2015; Gong et al., 2018), which potentially further weakens the leaf N-precipitation relationship. Meanwhile, consistent with some of previous findings that leaf N/P is determined largely by leaf P (Güsewell, 2004; Reich and Oleksyn, 2004; Ordoñez et al., 2009; Chen et al., 2013), our results demonstrate higher correlation of leaf N/P with leaf P than leaf N in their response to climate factors, implying a more important role of P in regulating leaf biochemical processes. Most notably, leaf N/P increases with increasing temperature or decreasing precipitation and humidity, suggesting high N concentration in leaf in high-temperature to enhance plant vegetative growth (due to high photosynthetic rate) for strengthening its individual competition (Davis et al., 1999; Gong et al., 2018), or high P allocation to reproductive structures in stressful low-moisture for promoting plant flowering and seed development (P is an essential element for plant sexual reproduction; Kerkhoff et al., 2006; Fujita et al., 2014).

Consistent with our third expectation and the common findings (Niinemets, 2001; Wright et al., 2005; Yang et al., 2019), plants adapt to the climate by the covariation of leaf traits. Obviously, two sets of leaf trait combination can be extracted from our RDA. The first trait set, main including leaf/stomata size traits (LA, LL, and SA), is observed to increase with temperature dimension, supporting that the effect of climate on leaf trait variation is primarily due to the constraint of heat quantity on the both cell- and organ-level leaf size development. The second trait set, main including water use-related leaf traits (e.g., SLA and SD), is observed to vary significantly with precipitation dimension, representing that the effect of climate on leaf trait variation is secondly caused by a water-limitation on leaf physiological processes. Our RDA also shows a negative association of leaf N/P with precipitation, and a positive association of leaf N with humidity and sunshine hour, but their weak association or the lack of covariation among multiple traits suggests that they seldom form trait syndrome in response to environments coordinatedlly.

## Conclusion

In line with our expectation, temperature is the most important climate factor affecting most of leaf traits, especially leaf chemical traits and leaf/stomata size and shape, suggesting a significant heat limitation on leaf development and physiological and biochemical processes in the cool QTP zone. Precipitation/humidity and sunshine hour influence mainly water use-related leaf traits and leaf/stomata size and shape, respectively. Besides, the RDA shows two sets of significant leaf trait-climate relationship, i.e., leaf/stomata size traits co-varying with temperature as well as water use-related leaf traits co-varying with precipitation, implying the substantial predictive power of the values of these traits in examining leaf responses to climate.

## Data Availability Statement

The original contributions presented in the study are publicly available. This data can be found here: NCBI repository, accession: PRJNA707202.

## Author Contributions

WQ and XK conceived the ideas. All authors collected the data. XK and JZ analyzed the data. XK, YL, and WQ wrote the manuscript. All authors read, commented on, and approved this version of the manuscript.

## Conflict of Interest

The authors declare that the research was conducted in the absence of any commercial or financial relationships that could be construed as a potential conflict of interest.

## Publisher’s Note

All claims expressed in this article are solely those of the authors and do not necessarily represent those of their affiliated organizations, or those of the publisher, the editors and the reviewers. Any product that may be evaluated in this article, or claim that may be made by its manufacturer, is not guaranteed or endorsed by the publisher.

## Footnotes

1
http://rsb.info.nih.gov/ij

2
http://cdc.cma.gov.cn

## Supplementary Material

Supplementary Appendix 1 Results of principal component analysis (PCA) and the correlation matrix of climatic variables.

Supplementary Data The location and climate attributes for 641 woody plant populations and their leaf traits.
