# Supplementary material for: Response of Leaf Traits of Eastern Qinghai-Tibetan Broad-Leaved Woody Plants to Climatic Factors
Source: Front Plant Sci. 2021 Jul 30;12:679726. doi: 10.3389/fpls.2021.679726 (PMC8363248; doi:10.3389/fpls.2021.679726)
Supplement: Supplementary Appendix 1 — Results of principal component analysis (PCA) and the correlation matrix of climatic variables. [file Table_1.DOCX]

### Results of principal component analysis (PCA) and the correlation matrix of environmental variables

PCA on eight climate variables showed that the first three (four) PC axes explained > 90% (> 95%) variation in the data, in which the PC 1, 2, 3 and 4 accounted for 43.978%, 36.731%, 11.692% and 4.673% of the total variance, respectively. The load of climate variables on the PC axes and their correlation matrix were seen in Table S1 and Table S2, respectively.

**Table S1.** The load of climate variables on the first four PC axes. The variables with the largest load on each PC axis were marked in bold.

| Climate variables | PC 1 | PC 2 | PC 3 | PC 4 |
| --- | --- | --- | --- | --- |
| Mean annual temperature (MAT) | 0.506 | 0.157 | NA | 0.200 |
| Mean temperature of warmest quarter (MTWQ) | **0.524** | NA | 0.123 | 0.162 |
| Mean temperature of coldest quarter (MTCQ) | 0.436 | 0.313 | NA | 0.270 |
| Mean annual precipitation (MAP) | -0.289 | 0.452 | 0.206 | NA |
| Precipitation of wettest quarter (PWQ) | -0.322 | 0.424 | -0.252 | 0.171 |
| Precipitation of driest quarter (PDQ) | -0.162 | **0.508** | NA | 0.356 |
| Mean annual sunshine hours (MASH) | -0.151 | -0.447 | -0.311 | **0.795** |
| Mean relative humidity (MRH) | -0.208 | -0.180 | **0.879** | 0.263 |

**Table S2.** The correlation matrix among eight climate variables. The strong correlations (r ≥ 0.7) were marked in bold.

|  | MAT | MTWQ | MTCQ | MAP | PWQ | PDQ | MASH | MRH |
| --- | --- | --- | --- | --- | --- | --- | --- | --- |
| MAT | - | **0.981** | **0.944** | -0.275 | -0.383 | -0.036 | -0.442 | -0.355 |
| MTWQ | <0.001 | - | **0.866** | -0.442 | -0.541 | -0.191 | -0.337 | -0.294 |
| MTCQ | <0.001 | <0.001 | - | 0.006 | -0.078 | 0.219 | -0.559 | -0.452 |
| MAP | 0.061 | 0.002 | 0.967 | - | **0.850** | **0.782** | -0.467 | 0.141 |
| PWQ | 0.008 | <0.001 | 0.602 | <0.001 | - | **0.793** | -0.261 | -0.170 |
| PDQ | 0.809 | 0.198 | 0.139 | <0.001 | <0.001 | - | -0.498 | -0.105 |
| MASH | 0.002 | 0.020 | <0.001 | 0.001 | 0.076 | <0.001 | - | 0.169 |
| MRH | 0.014 | 0.045 | 0.001 | 0.345 | 0.254 | 0.483 | 0.257 | - |

According to our variable selection criteria, MTWQ, PDQ and MRH were initially selected because their largest load on PC1, 2 and 3, respectively. We then removed MAT and MTCQ for they being strong correlated with MTWQ, and removed MAT and PWQ for they being strong correlated with PDQ. Finally, MASH was kept for it being less explained by other selected variables as well as its largest load on PC4.
